# Supplementary material for: Dynamic persistence of UPEC intracellular bacterial communities in a human bladder-chip model of urinary tract infection
Source: eLife. 2021 Jul 5;10:e66481. doi: 10.7554/eLife.66481 (PMC8354636; doi:10.7554/eLife.66481)
Supplement: Transparent reporting form [file elife-66481-transrepform.docx]

***eLife’s* transparent reporting form**

We encourage authors to provide detailed information *within their submission* to facilitate the interpretation and replication of experiments. Authors can upload supporting documentation to indicate the use of appropriate reporting guidelines for health-related research (see [EQUATOR Network](http://www.equator-network.org/%20)), life science research (see the [BioSharing Information Resource](https://biosharing.org/)), or the [ARRIVE guidelines](http://www.plosbiology.org/article/info:doi/10.1371/journal.pbio.1000412) for reporting work involving animal research. Where applicable, authors should refer to any relevant reporting standards documents in this form.

If you have any questions, please consult our Journal Policies and/or contact us: [editorial@elifesciences.org](mailto:editorial@elifesciences.org).

**Sample-size estimation**

- You should state whether an appropriate sample size was computed when the study was being designed
- You should state the statistical method of sample size computation and any required assumptions
- If no explicit power analysis was used, you should describe how you decided what sample (replicate) size (number) to use

Please outline where this information can be found within the submission (e.g., sections or figure legends), or explain why this information doesn’t apply to your submission:

Our infection protocol was optimized to generate <1 foci of infection per epithelial cell. On each infected chip, we imaged between 25-50 fields of view, each 206 x 206 μm^2^ corresponding to most of the surface area of the chip. The number of fields of view imaged was restricted by the temporal resolution of image acquisition, and so was lower in experiments that studied the dynamics of neutrophil migration. Wherever possible, data was obtained from at least n=3 independent bladder-chips. Owing to experimental challenges, it was not possible to track the evolution and fate of every IBC on-chip. We performed a sufficient number of experiments to capture the bacterial growth dynamics in n=161 individual IBCs, and report on the eventual fate of n=100 individual IBCs. This provided us the ability to detect phenotypes with a relatively low frequency of occurrence (~1-2%). However, the cell fate phenotypes of interest to us in this study viz. cell exfoliation and/or bacterial shedding were observed with a much higher frequency. Within the overall population of IBCs studied, a small number of IBCs (n=18) were successfully tracked over two rounds of antibiotic treatment. Data from this sub-population was used to confirm that phenotypes such as bacterial regrowth upon antibiotic removal or the absence of elimination of bacteria within IBCs by the antibiotic treatment could occur, but was not used for statistical comparisons.

**Replicates**

- You should report how often each experiment was performed
- You should include a definition of biological versus technical replication
- The data obtained should be provided and sufficient information should be provided to indicate the number of independent biological and/or technical replicates
- If you encountered any outliers, you should describe how these were handled
- Criteria for exclusion/inclusion of data should be clearly stated
- High-throughput sequence data should be uploaded before submission, with a private link for reviewers provided (these are available from both GEO and ArrayExpress)

Please outline where this information can be found within the submission (e.g., sections or figure legends), or explain why this information doesn’t apply to your submission:

Information concerning the number of independent bladder-chips used (biological replicates), the number of fields of view sampled on each bladder-chip (technical replicates) and the sample sizes are indicated in the Figure and Figure Supplement Legends.

**Statistical reporting**

- Statistical analysis methods should be described and justified
- Raw data should be presented in figures whenever informative to do so (typically when N per group is less than 10)
- For each experiment, you should identify the statistical tests used, exact values of N, definitions of center, methods of multiple test correction, and dispersion and precision measures (e.g., mean, median, SD, SEM, confidence intervals; and, for the major substantive results, a measure of effect size (e.g., Pearson's r, Cohen's d)
- Report exact p-values wherever possible alongside the summary statistics and 95% confidence intervals. These should be reported for all key questions and not only when the p-value is less than 0.05.

Please outline where this information can be found within the submission (e.g., sections or figure legends), or explain why this information doesn’t apply to your submission:

All information regarding the statistical tests used, the exact N values, mean and confidence interval and the P values are listed in the Figure Legends, where appropriate in the main text and within the Figures 1-4 in the main text and Figure Supplements.

(For large datasets, or papers with a very large number of statistical tests, you may upload a single table file with tests, Ns, etc., with reference to sections in the manuscript.)

**Group allocation**

- Indicate how samples were allocated into experimental groups (in the case of clinical studies, please specify allocation to treatment method); if randomization was used, please also state if restricted randomization was applied
- Indicate if masking was used during group allocation, data collection and/or data analysis

Please outline where this information can be found within the submission (e.g., sections or figure legends), or explain why this information doesn’t apply to your submission:

Group allocation was not relevant for our submission.

**Additional data files (“source data”)**

- We encourage you to upload relevant additional data files, such as numerical data that are represented as a graph in a figure, or as a summary table
- Where provided, these should be in the most useful format, and they can be uploaded as “Source data” files linked to a main figure or table
- Include model definition files including the full list of parameters used
- Include code used for data analysis (e.g., R, MatLab)
- Avoid stating that data files are “available upon request”

Please indicate the figures or tables for which source data files have been provided:

Source data for Figures 1 – 4 and the accompanying Figure Supplements have been uploaded to the EPFL community pages at Zenodo and are available at the following doi: 10.5281/zenodo.5028262
